# A database of heterogeneous faces for studying naturalistic expressions

**DOI:** 10.1038/s41598-023-32659-5

**Published:** 2023-04-03

**Authors:** Houqiu Long, Natalie Peluso, Chris I. Baker, Shruti Japee, Jessica Taubert

**Affiliations:** 1 The School of Psychology, The University of Queensland, St Lucia, QLD Australia; 2 Laboratory of Brain and Cognition, National Institute of Mental Health, Bethesda, MD USA

**Keywords:** Psychology, Human behaviour

## Abstract

Facial expressions are thought to be complex visual signals, critical for communication between social agents. Most prior work aimed at understanding how facial expressions are recognized has relied on stimulus databases featuring posed facial expressions, designed to represent putative emotional categories (such as ‘happy’ and ‘angry’). Here we use an alternative selection strategy to develop the Wild Faces Database (WFD); a set of one thousand images capturing a diverse range of ambient facial behaviors from outside of the laboratory. We characterized the perceived emotional content in these images using a standard categorization task in which participants were asked to classify the apparent facial expression in each image. In addition, participants were asked to indicate the intensity and genuineness of each expression. While modal scores indicate that the WFD captures a range of different emotional expressions, in comparing the WFD to images taken from other, more conventional databases, we found that participants responded more variably and less specifically to the wild-type faces, perhaps indicating that natural expressions are more multiplexed than a categorical model would predict. We argue that this variability can be employed to explore latent dimensions in our mental representation of facial expressions. Further, images in the WFD were rated as less intense and more genuine than images taken from other databases, suggesting a greater degree of authenticity among WFD images. The strong positive correlation between intensity and genuineness scores demonstrating that even the high arousal states captured in the WFD were perceived as authentic. Collectively, these findings highlight the potential utility of the WFD as a new resource for bridging the gap between the laboratory and real world in studies of expression recognition.

## Introduction

Humans are deeply social beings. We thrive on sharing our needs, wants, and feelings with those around us, in part through our facial expressions. Expression recognition has attracted the attention of researchers in multiple fields because impairments in this ability are associated with major neuropsychological conditions including autism^1,2^, bipolar disorder^3^, schizophrenia^4^, and cognitive decline^5^. Further, with the rise of video calls, online learning, remote work, artificial intelligence, and virtual reality, it has become increasingly important to understand how we recognise expressions in digital social agents and in online tasks^6,7^. However, despite its importance, our understanding of expression recognition is constrained by the kinds of staged, artificial stimuli researchers have selected for use in previous experimental tasks. Here we develop an alternative method for stimulus selection, producing a database of ambient facial expressions that reflect the diverse conditions under which we naturally see faces.

Our current understanding of how we perceive and recognize facial expressions has relied heavily on the use of posed facial expressions, taken in sterile laboratory conditions, because researchers have prioritized the control of other facial properties and low-level visual attributes^8–12^. For example, researchers have used photographs of actors asked to convey different facial expressions because this allowed them to control other image properties such as facial identity, gaze direction, viewing angle, lighting conditions and the presence of background cues^13–15^. This method of stimulus creation and selection has allowed researchers to develop experimental tasks to tap into expression recognition, while minimising interference from other image properties, context or familiarity with individual actors. However, the validity of this approach depends on a longstanding theory that there are “basic” facial expressions that are important visual signals linked to putative emotions and internal states with biological and evolutionary origins^16,17^; a theory that is being challenged^18–22^. The central debate is focused on whether there is a set of universally recognized facial expressions that occur spontaneously outside of the laboratory^23–26^. Consequently, it is possible that face stimuli created to represent semantic emotional categories such as “happy”, “angry” and “fearful” might underestimate or poorly characterize the psychometric space that underlies our capacity for expression recognition in everyday life.

There is also emerging evidence that humans are aware of the artificial nature of posed facial expressions^27^ and that this awareness might alter behavioral responses to those expressions^28^ and the associated patterns of brain activity^29^. Therefore, to advance our understanding of expression recognition we must make a genuine effort to increase ecological validity and use more naturalistic facial expressions in experiments. Previous reports of such efforts to gather naturalistic stimuli include databases such as the Aff-Wild2^30^ which is a large database of dynamic faces classified on the basis of muscle movements. In contrast, our goal was to provide a database of one thousand static images depicting ambient facial behaviors and employ a data-driven approach to describe the emotional content. This database is hereafter referred to as the Wild Faces Database or the WFD.

This project proceeded in two stages. First, we conducted a large-scale web-based search for images and videos of faces. This involved multiple researchers starting with a set of search terms to guide the collection of images and videos with appropriate usage rights from online platforms. Several exclusion criteria were employed to remove famous identities and poor-quality images from the WFD (see “Materials and methods” for more details). Thus, the WFD contains 840 human faces (415 feminine in appearance) representing a wide range of ages, races, and ethnicities, as well as 101 animal faces and 59 illusory faces in objects. Illusory faces were included because the perception of faces in non-face objects—a phenomenon known as face pareidolia—is thought to emerge from our motivation for social connectivity^31–36^. As such, images inducing pareidolia may provide an entry point for research exploring shared mechanisms between real faces and objects that simply look face-like^37,38^, and for exploring bias towards other attributes like perceived gender in expression perception^36^. The inclusion of animal expressions (the majority being non-human primates) might also prove vital when considering the developmental origins of facial signals in terms of both production and recognition^39–42^.

In the second stage of this project, we used a data-driven approach to characterize the perceived emotional content of the WFD images. To evaluate the utility of the selection method, we also compared the WFD images to (1) one thousand images taken from existing and conventional facial expression databases, and (2) one thousand images taken from the 10 k Adult Faces Database^40^. In addition to categorizing the facial expression in each image, participants were asked to rate the intensity and genuineness of the expression. The results revealed that images in the WFD elicited more variable and less specific responses from participants in terms of emotional categorization, compared to images taken from other databases. This increased variability in the response profile of WFD images might help contextualize more fine-grained, socio-emotional content present in facial behaviors in everyday life. WFD images were also rated as *less* intense and *more* genuine than images taken from other databases. In sum, the Wild Faces Database is a large, diverse, and contextually rich resource made publicly available via the Open Science Framework (https://osf.io/6p4r7/).

## Results

**Figure 1:**
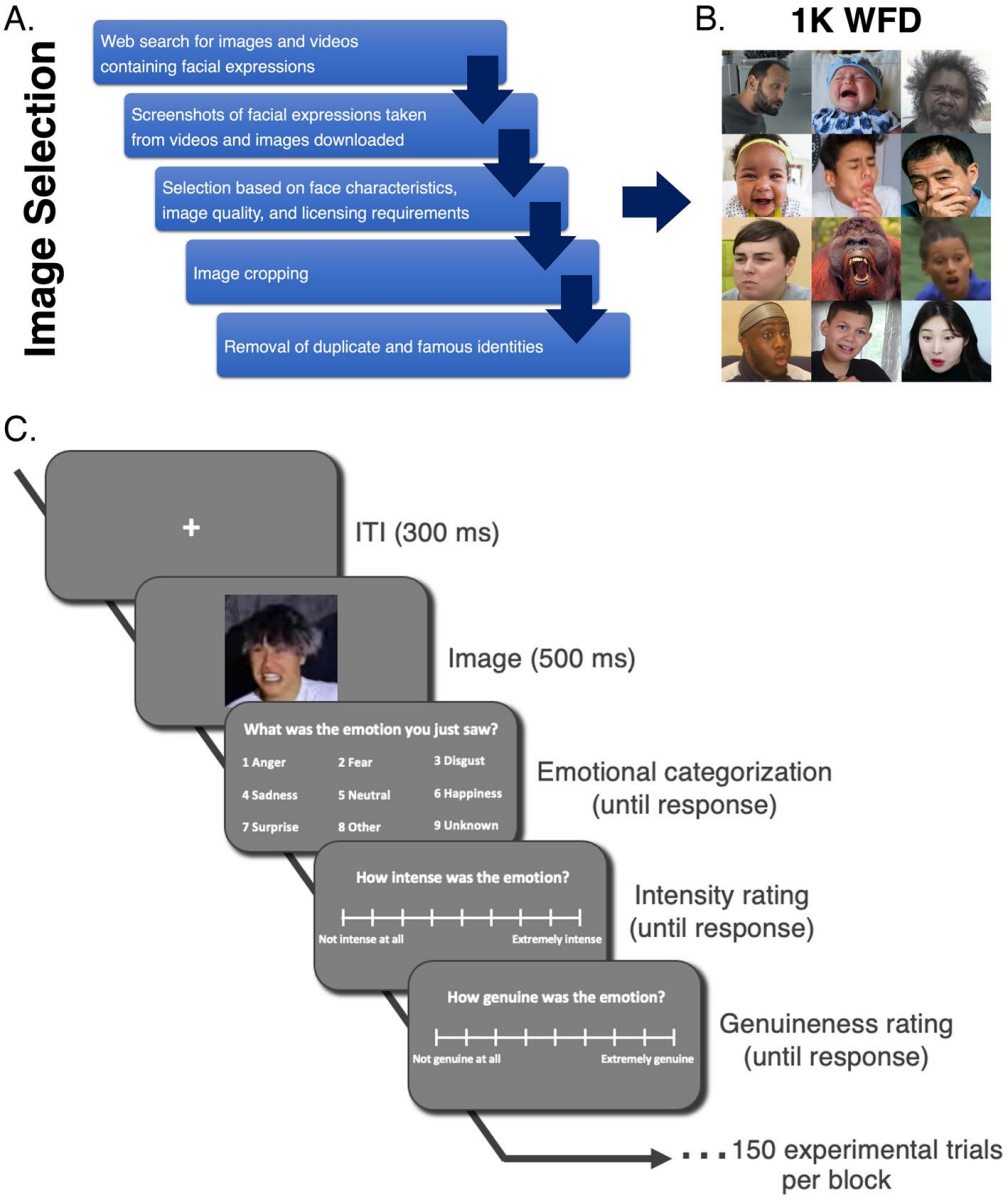
(**A**) Schematic showing the procedure used to identify and select the WFD faces. (**B**) Illustrative examples of the diverse images included in the WFD. (**C**) The trial procedure for the behavioral ratings experiment. On each trial, participants were shown an image for 500 ms followed by a prompt to categorize the emotion displayed in the image using one of nine options. This was followed by prompts to obtain intensity and genuineness ratings using a 9-point scale. Each participant completed 50 WFD trials, 50 ED trials and 50 US trials per block (each participant viewed 1–3 unique blocks).

To create the WFD, we performed an ‘intelligent scrape’ of the public domain for one thousand images of naturalistic facial behaviors (see Fig. 1A). Specifically, three authors (H.L., N.P., and J.T.), working initially independently and then collectively, used a large variety of emotional search terms (such as “best day ever” and “outburst”; see Table 1 for a catalogue of these terms) to identify candidate images. These search terms were inspired by the basic facial expressions, to try and capture a wide range of emotions, but these terms did not equally contribute to the database and often the authors had to follow sub-threads or use combinations of terms to find candidate images. Importantly, even though we documented this process, at no point were the search terms considered to be a ground truth nor do we believe these search terms should be used as the “correct” labels.

**Table 1:** Examples of WFD stimuli search terms.

	Anger	Fear	Disgust	Sadness	Happy	Surprise
1	Angry	Scare	Disgust	Sad	Happy	Surprise
2	Angry reaction	Scared reaction	Disgusting reaction	Sad reaction	Happy reaction	Surprised reaction
3	Never felt so angry	Don’t fear challenge	Disgusted food	Crying	Best day ever	Shock reaction
4	So furious	Scare challenge reaction	Food tasting	Crying reaction	Laugh	Shocking faces
5	Mad	Fear reaction	So revolting	Made me cry	Laugh challenge	Shocking Asian reactions
6	I get so angry when	Fear face	Don’t look away challenge	Upset	Happy couple	So surprised
7	Hate it	Scariest moments	Gross food	Loss	Happiest day	Cheating caught
8	Losing my temper	Horror film	Food challenge	Depression	Wedding	Cheating reaction
9	Outburst	Shooting	Food tasting	Crying reaction	Propose	Couldn’t believe it
10	Losing their cool	Ghost videos	Worst smells	Don’t cry challenge	Funny videos	Wasn’t expecting that

Next images were cross-checked for visual quality and permission for reuse with modification. This selection method resulted in a highly heterogeneous collection of visual stimuli, depicting a wide range of people, animals and objects in naturalistic contexts and photographed or filmed using different cameras and equipment (see Fig. 1B for illustrative examples). Having collected these images, we focused on two key empirical questions: (1) does this selection method yield images representing a range of different recognizable facial expressions? And (2) how does this stimulus selection method compare to other selection methods?

To address these questions, we compared the thousand images in the WFD to one thousand images taken from existing databases of facial expressions (referred to as the ‘Existing Database’ or ED faces), and another thousand images selected at random from the *10 k US Adult Faces Database*^43^ (referred to as the ‘Unbiased Selection’ or US faces). For more detailed information about stimulus sources see “Materials and methods”. The purpose of this comparison was to evaluate the utility of the WFD against two common practices in studies of expression recognition. For example, researchers often download and use highly controlled stimuli from existing databases, an approach represented by the ED faces in this experiment. In contrast, an equally valid but opposing approach is to program a machine to scrape images from the public domain without imposing any further selection criteria. This approach is represented by the US faces in this experiment, which were selected to reflect different identities, not expressions. While both approaches have their distinct advantages, our expectation is that the WFD faces will provide researchers with a complementary “middle ground”, with more ecological validity than the ED faces and a more diverse range of recognizable emotional content than the US faces.

### Emotion categorization

**Figure 2:**
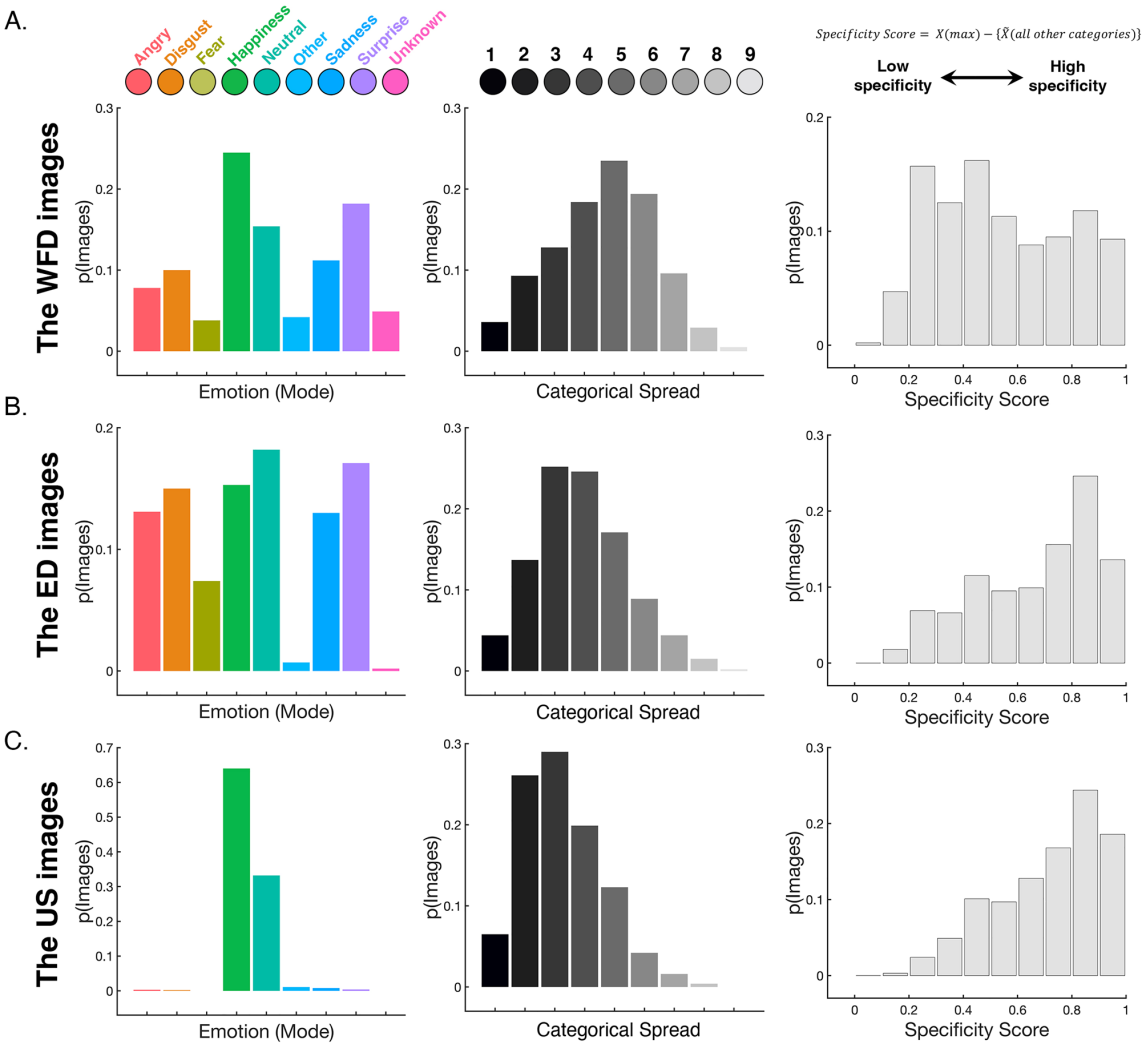
Results of the emotion categorization task. The distribution of modal scores (left), categorical spread scores (middle), and specificity scores (right) for images in the (**A**) Wild Faces Database (WFD images), (**B**) Existing Databases (ED images) and (**C**) the Unbiased Selection (US images). The WFD images showed higher categorical spread in how participants perceived the expressions compared to the ED and US images. ED and US images expectedly showed greater specificity compared to WFD images.

Each of the 3000 images (1000 images each from WFD, ED and US faces) was categorized by 18–22 participants (for details of the experimental procedure see Fig. 1C). We calculated the modal score for the perceived emotional expression of each image. For the WFD faces (anger: 7.8%, disgust: 10%, fear: 3.8%, happiness: 24.5%; neutral: 15.4%, sadness: 11.2%, surprise: 18.2%, other: 4.2%, unknown: 4.9%, Fig. 2A) and the ED faces (anger: 13.1%, disgust: 15%, fear: 7.4%, happiness: 15.3%, neutral: 18.2%, sadness: 13%, surprise: 17.1%, other: 0.7%, unknown: 0.2%; Fig. 2B) a broad range of emotions were perceived across the different images. In contrast, the distribution of modal responses for the US faces was more restricted and biased towards neutral and happy expressions (anger: 0.3%, disgust: 0.2%, fear: 0%, happiness: 64%, neutral: 33.2%, sadness: 0.8%, surprise: 0.4%, other: 1.1%, unknown: 0%; Fig. 2C). This is consistent with the notion that unbiased sampling of the internet might oversample a narrow range of emotional content because people will be motivated to upload more attractive and approachable impressions of themselves (i.e., photographs of themselves looking happy or emotionally neutral rather than aggressive or fearful) into the public sphere^44–49^.

We also examined the spread in responses across participants by calculating the number of perceived emotional expressions used to categorize each image. Thus, if an image had a categorical spread score of 1, it meant that all the participants used the same option to categorize the image. Alternatively, if an image had a categorical spread score of 9, it meant that participants used all nine options to categorize the image. We found that the distribution of categorical spread scores for the WFD faces (*median* = 5, *range* = 1–9; Fig. 2A) was different from the distribution of spread scores for the ED faces (*median* = 4, *range* = 1–9, Fig. 2B; *Mann–Whitney U test, Z* = − 10.38, *p* < 0.001, *two-tailed*) and the US faces (*median* = *3*, range = 1–8, Fig. 2C; *Mann–Whitney U test,* Z = − 18.24, p < 0.001, *two-tailed*). In addition, to summarize and compare the specificity of responses across databases, for each image we calculated a specificity score by subtracting the average frequency to all other emotion categories from the frequency associated with the modal emotion category (Fig. 2A–C). When we compared the WFD to the other databases, we found that the distribution of specificity scores for the WFD faces (*median* = 0.52, *range* = 0.09–1; Fig. 2A) was different from the distribution of specificity scores for the ED faces (*median* = 0.72, *range* = 0.11–1, Fig. 2B; *Mann–Whitney U test, Z* = − 11.34, *p* < 0.001, *two-tailed*) and the US faces (*median* = 0.76, range = 0.11–1, Fig. 2C; *Mann–Whitney U test,* Z = − 15.87, p < 0.001, *two-tailed*). The direction of the differences in both spread and specificity indicates that faces in the WFD elicited more variable responses than faces in the ED or US databases.

What is the source of this variability? It could be that the higher degree of response variability reflects increased task difficulty; the facial expressions in the WFD images are noisier visual signals than those in the ED or US images and are, therefore, more difficult to recognize and elicit more random guesses. To examine this possibility, we ran a split-half reliability analysis and compared the three databases. If the variability in responses to the WFD faces reflects random guessing, we would not expect any correlation in the responses across groups of participants. However, if the variability reflects a true ambiguity in the expression, we would expect the responses to the WFD faces to correlate as strongly as for the US and ED faces. To do this, we sorted the participants into two groups based on their randomly assigned participant numbers (odd numbers vs. even numbers) and then, for every image in the 3 database conditions, we calculated a separate specificity score for the odd and even groups. The Pearson correlation between the scores given by the two groups for the images yielded an *r* of 0.69 for the WFD (*N* = 1000, *p* < 0.001, *two-tailed*), 0.68 for the ED (*N* = 1000, *p* < 0.001, *two-tailed*) and 0.6 for the USD (*N* = 1000, *p* < 0.001, *two-tailed*). The statistical significance of these tests indicates that responses were reliable and not noisy. When we compared the strength of these correlations on a pairwise basis, we found no evidence of a difference between the WFD and the ED (*z* = 0.42, *p* = 0.337). The comparable strength of these correlations suggests that the responses towards the WFD images were as reliable across participants as the responses towards the posed facial expressions in the ED. The pairwise comparisons also found evidence of less reliability among responses to the US database than responses to the other databases (USD vs. WFD, *z* = 3.46, *p* < 0.001; USD vs. ED, *z* = 3.04, *p* = 0.001). Further when we computed the mode emotional category for the WFD faces separately for the odd and even groups, we found that a large proportion of them (79.4%) had the same mode regardless of group. Collectively, these results suggest that increased task difficulty and visual noise are insufficient explanations for the degree of categorical spread and specificity associated with the WFD.

**Figure 3:**
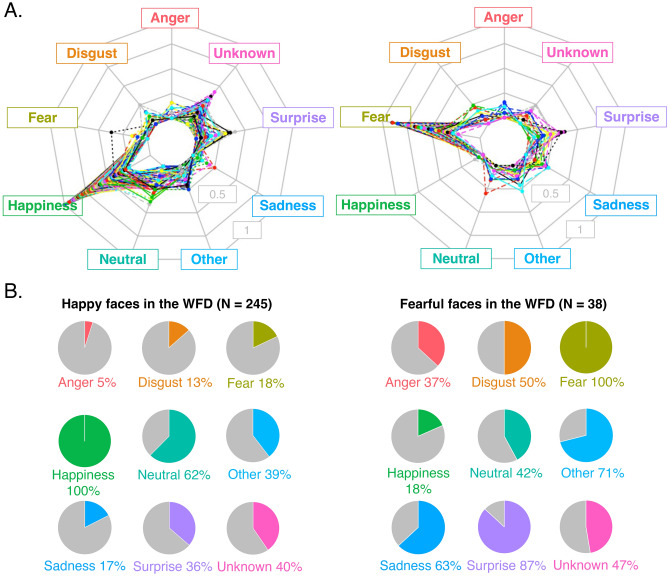
Not all happy and fearful faces are created equal. (**A**) Radial plots showing the response rates for each emotion category for WFD faces that were most frequently categorized as expressing “happiness” (245 images; left) and “fear” (38 images; right). Note that even amongst these top ‘happy’ and ‘fearful’ faces, participants picked other responses to categorize the same expression. (**B**) Pie charts showing the proportion of the 245 happy (left) and 38 fearful faces (right) that were categorized using each of the other emotion categories. For example, while *all* of the 245 happy faces were categorized by at least one participant as signalling happiness (by definition), 13% of the happy faces were categorized by at least one participant as signalling disgust, and 36% of the happy faces were categorized by at least one participant as signalling surprise. Similarly, while *all* of the 38 fearful faces were categorized by at least one participant as signalling fear, 18% were categorized as happy and more than half (i.e., 71%) were categorized as signalling a recognizable but unspecified emotion (i.e., “other”).

**Figure 4:**
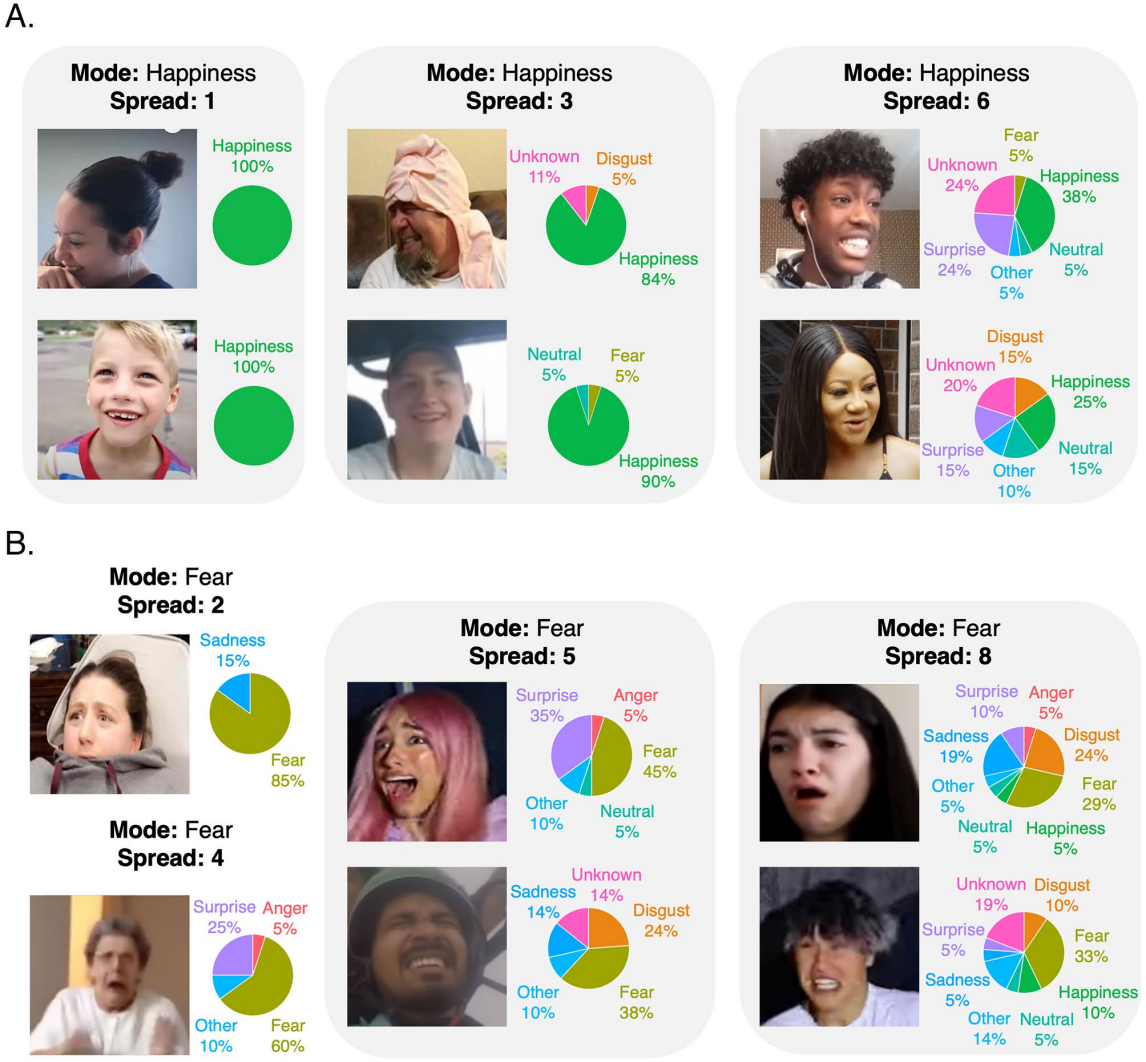
Six representative examples of WFD faces with modal scores of happy (**A**) and fearful (**B**). From left to right the examples vary in their categorical spread from low to high. Each image was categorized by 18–22 participants. The pie charts show the distribution of categorical responses for each image.

Next, we considered the possibility that the reason the WFD faces elicited more variable responses from the participants than the ED or US faces, was because more naturalistic facial behaviours transmit composite signals that are better characterized by using multiple emotional tags. For example, in Fig. 3 we took a detailed look at the responses to the WFD faces that were most frequently rated as expressing happiness or fear. Studies of happy faces that have routinely reported variance in the use and meaning of a smile^50–52^ and despite being an “opposing” emotional category associated with negative valence, and occurring at a lower frequency than happiness in the WFD than happy expressions (see Fig. 2A), similar questions have been raised about the variable meaning of fearful facial expressions^53,54^. Thus*,* these are two emotional categories for which there is an interest in interpreting variance in participant responses. What we discovered was that, although the response profile of these faces was dominated by “happiness” and “fear”, respectively, these faces also elicited distinct combinations of other emotional responses (Fig. 3A,B). Indeed, summarizing each image using multiple emotional dimensions seemingly captures more fine-grained, diagnostic, information (see Fig. 4A,B). Thus, modal, spread and specificity scores provide independent, yet equally important, descriptions of emotional content.

### Intensity ratings

**Figure 5:**
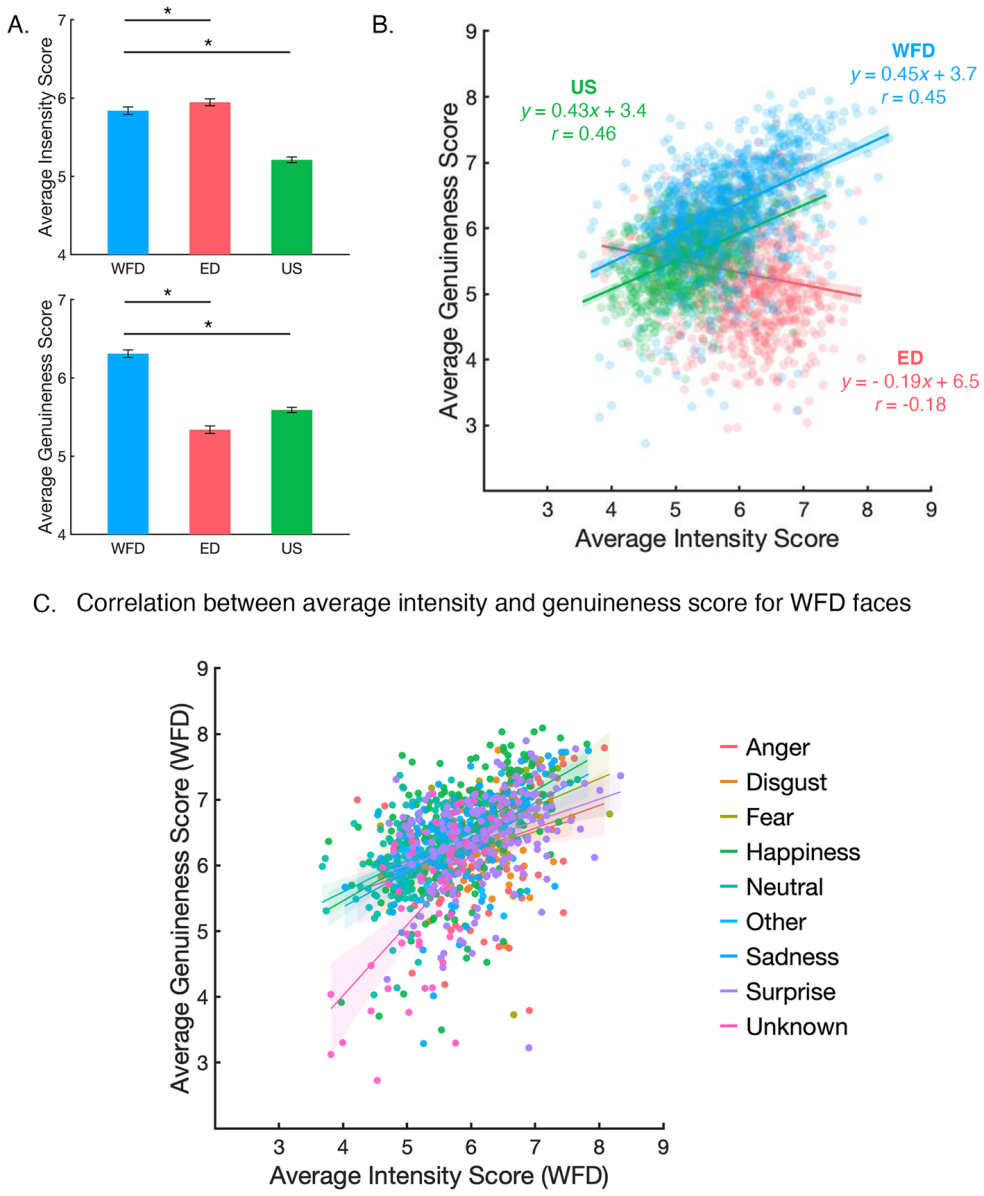
(**A**) Average expression intensity (top) and genuineness (bottom) scores as a function of the three databases (WFD, ED, and US). Error bars are 95% *CI*. Lines with asterisks indicate significant pairwise differences (*p* < 0.001). (**B**) Average intensity scores were highly correlated with genuineness scores for images in the WFD (blue markers), ED (red markers), and US (green markers) databases. The corresponding solid lines are the result of the best fitting linear regression of the form *y* = *mx* + *b*. (**C**) The relationship between genuineness and intensity for the WFD redrawn to examine the influence of modal categorization. Color reflects the emotion most often used by human participants to categorize the image. The solid lines are the result of the best fitting linear regression, as in (**B**), and their length marks the data range. Thus, any clustering of colored dots would indicate that particular emotions are perceived as more genuine and/or intense than others. However, this is not the case, all colors are dispersed, and we observe positive relationships between genuineness and intensity for every emotional category. That said, this plot does not take into account categorical spread and specificity score.

A mean expression intensity score was calculated for each of the 3000 images. A one-way, independent samples ANOVA was performed to compare average intensity across the three databases. The results of this analysis revealed a significant effect of database, *F*(2, 2997) = 323.9, *p* < 0.001, *ƞ*^2^_*p*_ = 0.18 (Fig. 5A). We used two a-priori contrasts to determine whether the intensity of the WFD differed from the other two databases. The first of these contrasts revealed that the expressions of the WFD faces (*M*_*WFD*_ = 5.84, *SE* = 0.02) were perceived as significantly *less* intense than the expressions of the ED faces (*M*_*ED*_ = 5.95, *SE* = 0.02; *independent samples t-test*, *t*(1998) = 3.22, *p* < 0.001, Cohen’s *d* = 0.14, *two-tailed*). In contrast, the expressions of the WFD faces were perceived as significantly *more* intense than the expressions of the US faces (*M*_*US*_ = 5.21, *SE* = 0.02; *independent samples t-test*, *t*(1998) = − 20.33, *p* =  < 0.001, Cohen’s *d* = − 0.91, *two-tailed*).

### Genuineness ratings

The mean genuineness ratings of the expressions were also calculated for all images. Again, a one-way, independent samples ANOVA was performed to compare average genuineness across the three databases. The results revealed a significant effect of database, *F*(2, 2997) = 517.15, *p* < 0.001, *ƞ*^2^_*p*_ = 0.26 (Fig. 5A). To test whether the WFD faces were rated as more genuine than faces in other databases, we performed two comparisons which revealed that the emotional expressions in the WFD faces (*M*_*WFD*_ = 6.31, *SE* = 0.02) were rated as significantly *more* genuine than the emotional expressions in the ED faces (*M*_*ED*_ = 5.34, *SE* = 0.02; *independent samples t-test*, *t*(1998) = − 28.3, *p* < 0.001, Cohen’s *d* = − 1.27, *two-tailed*) or US faces (*M*_*US*_ = 5.59, *SE* = 0.02; *independent samples t-test*, *t*(1998) = − 24.10, *p* =  < 0.001,Cohen’s *d* = − 1.08, *two-tailed*).

### The relationship between intensity and genuineness

For the images in each database (WFD, ED, and US faces) we examined the relationship between mean intensity and genuineness scores. For the WFD images we found a significant positive relationship between the two variables (*N*_*WFD*_ = 1000, *Pearson’s r* = 0.45, *p* < 0.001; Fig. 5B), indicating that the more intense an expression was perceived to be, the more genuine it was perceived to be as well. This was also evident for images in the unbiased selection (*N*_*US*_ = 1000, *Pearson’s r* = 0.46, *p* < 0.001; Fig. 5B). However, surprisingly, we found evidence of a significant negative relationship between intensity and genuineness scores for the faces from existing databases (*N*_*ED*_ = 1000, *Pearson’s r* = − 0.18, *p* < 0.001; Fig. 5B), indicating that the more intense an expression was perceived to be, the less genuine. This is consistent with the view that posed expressions might exaggerate signals beyond normal limits—to the point where they are perceived as disingenuous. To examine whether modal score predicted intensity and/or genuineness, we redrew the correlation between mean intensity and genuineness scores for the WFD in Fig. 5C. We found no evidence that particular facial behaviors, such as those most frequently categorized as happy, were perceived as more intense or more genuine than other facial behaviors.

## Discussion

In this paper our main goal was to develop and characterize a novel database of naturalistic images, representing the facial behaviors we tend to see in our everyday lives. To this end we collected 1000 images of faces and characterized the emotional content in each image using four dependent variables, including mode categorization, between-participant categorical spread, expression intensity and expression genuineness scores. We compared the WFD faces to two benchmarks: (1) a set of highly posed face stimuli, typically employed by researchers to control for low-level visual properties and (2) a set of faces scraped from the internet, typically employed by researchers to avoid selection biases. Our results support the notion that the WFD provides a complementary approach to the study of expression recognition; the WFD faces were perceived as *more* diverse in expressions than the images selected by means of an unbiased web-based selection (i.e., US faces) (Figs. 2, 3, 4) yet *more* genuine than photographs taken of facial expressions under more controlled circumstances (i.e., ED faces) (Fig. 5A). Finally, more emotional categories were used to describe the WFD faces than the ED or US faces (Fig. 2A), i.e., participants were less specific when responding to WFD faces. This observation suggests that naturalistic facial expressions, like the ones we see in everyday life, might be poorly characterized by basic-level category labels.

Unexpectedly, we found a negative relationship between average intensity and genuineness scores that was unique to faces selected from exiting databases (Fig. 5B). This finding suggests that when expressions are posed or generated under sterile laboratory conditions, the heightened intensity might decrease the expression’s perceived authenticity. Meanwhile, the opposite was true for the faces selected using web-based searches; for the WFD and US faces increases in intensity were associated with increases in authenticity. The simplest interpretation of these results is that posed expressions are noticeably exaggerated signals. Then again, although ED faces were rated as more intense than the WFD faces (Fig. 5A), the effect size was small and the ED faces tended to vary in terms of their average intensity score (see Fig. 5B). Thus, it is possible that the perceived genuineness of an expression is conveyed by more nuanced visual cues than those associated with intensity (see^27^). An important goal of future research will be to identify the factors that contribute to the perceived genuineness of a facial expression and see how they relate to other aspects of face evaluation. The negative relationship between intensity and genuineness for posed facial expressions could be related to the uncanny valley effect—where false or unusual visual cues in faces trigger an eeriness feeling in participants^55–58^. Perhaps staged or exaggerated facial expressions trigger similar mechanisms? Certainly, a deeper understanding of how humans detect false, disingenuous facial expressions is a topic for future research. Whether the ability to produce expressions under false pretences is linked to the ability to detect false expressions in others, and whether these abilities are unique to the human species^58,59^ are interesting questions that also follow from this line of thought.

A previous attempt to quantify the emotional content in naturalistic facial expressions produced outside of the laboratory used the movement of facial muscles to classify images^30^. This approach avoids the limitations associated with human selection and shows that machines can be trained to recognize the visual patterns that define canonical, basic facial expressions such as happiness. An added benefit of this approach is that the expressions are defined and annotated in dynamic displays, not just static images^30^. Motion has been identified as an important feature of facial expressions because they change over time^60–63^. However, a recent meta-analysis has indicated that there is no strong evidence linking the movement of particular muscles to discrete internal, affective states^21^. Other research has revealed that the basic categories often used to label facial expression do not transcend language and culture^18,22^. In sum, there is now speculation and considerable debate about how we should classify and label facial expressions. Therefore, here our central goal was to leverage a data-driven approach to shed light on the emotional content of naturalistic, ambient faces without making assumptions about correct labels and categories. Our experimental design allowed us to compare the static faces in the WFD to other static face stimuli, the results revealing that the WFD faces evoked more variable and less specific responses from participants than the ED or US faces. Whether adding motion to naturalistic visual stimuli would change behavioral and physiological responses remains to be seen.

Overall, the WFD provides a range of different naturalistic, ambient facial behaviors, with increased genuineness compared to other currently available resources. Close inspection of the WFD images provides evidence that naturalistic facial behaviors are multiplexed visual signals^26,64^; they tend to signal more than one basic emotion (see Fig. 3A). Contextualizing these latent dimensions will prove important for understanding how facial behaviors are read and recognized by both biological and artificial visual systems. We envision that researchers will sample images from the WFD based on their particular research questions. For example, the WFD allows for the comparison of specific versus ambiguous stimuli which provides new avenues for neuropsychological research (i.e., perhaps some patient groups respond abnormally to specific expressions owning to language difficulties whereas other groups respond abnormally to ambiguous expressions owning to limited social skills). Other researchers may need a selection of WFD images because their question requires ambient facial stimuli with high specificity and high genuineness. Aside from increased ecological validity and multidimensional granularity, the use of the WFD comes with limitations; for example, the WFD is not immune to the limitations associated with language because we used emotionally charged words and phrases in the search procedure. Thus, we expect that the WFD does not represent the entire spectrum of spontaneous human facial behaviors. For this reason, we hope that by making the WFD freely available to the scientific community, over time other groups can help develop and extend the WFD by adding new images found using different search terms and other resources. This will ultimately enable large-scale naturalistic research in the field of expression recognition.

## Materials and methods

All methods were performed in accordance with the relevant guidelines and regulations.

### Stimulus selection methods

#### Image selection for the Wild Faces Database

We gathered one thousand naturalistic images of facial behaviors, with appropriate Creative Commons licensing, using extensive web-based searches. To capture these spontaneous expressions in context, we searched videos hosted by YouTube (659 videos) and Chinese-hosted video channel Bilibili (69 videos). Once a suitable video was identified, the video was paused at an appropriate frame and screen snipped. The resulting image was cropped to 400 pixels × 400 pixels. We also sourced 157 images from Pxhere.com, and 25 from Pexels.com using the same criteria as above. Remaining images were photographs taken and belonging to one of the authors (J.T.). Searches were guided by seven basic emotion categories: happiness, sadness, anger, surprise, fear, disgust and neutral. This was to ensure a relatively good spread of possible facial behaviors. However, since we generated a list of search terms that were based on the basic emotion categories, this means that our selection process is still circumscribed.

No facial attributes were used as selection criteria except for familiar identities (which were excluded). Thus, the WFD contains images that vary in viewpoints, colour profiles, backgrounds, obstructions and/or clothing and make-up. Celebrities known to the authors were removed from the WFD but it remains possible that some faces are either highly memorable or will be known to others. Very few image properties were used as selection criteria—as long as the image or frame could be cropped into a square shape without transparent pixels, it was considered appropriate. Once resized to 400 pixels in width, we verified that the images could be seen on three different screens (including a Dell LCD monitor). This means that some of the faces in the WFD appear heavily pixelated, for example, or contain motion blur artifacts. Strong consideration was given to non-WEIRD (Western, Educated, Industrialized, Rich and Democratic) representation, as well as an inclusive spectrum of gender, age, and disability. Since the search terms in Table 1 often yielded animal faces, examples of face pareidolia and portraits, we included some examples of these in the WFD. That said, the exact proportion of non-human faces were not controlled in any strict way. The most appropriate emotion category was recorded along with its source, the assumed or explicitly stated gender category and assumed or explicitly stated age of the individual.

The final set of one thousand WFD images included a broad range of human and animal faces and face-like objects (840 human, 101 animals, and 59 objects) with human faces representing multiple races, different genders (415 feminine, 403 masculine, and 22 other), a diverse age range (36 infant, 99 child, 50 adolescent, 548 younger adults, and 107 older adults) and eight persons with a known disability (three blind, two Stromme Syndrome, one Cerebral Palsy, one Grayson’s Syndrome, and one Rett’s Syndrome). The WFD stimuli and corresponding data are available through the Open Science Framework at https://osf.io/6p4r7/.

#### Image selection for the Existing Database (ED) condition

To compare the WFD faces to more conventional stimuli, we selected an equal number of images from nine existing databases of emotional facial expressions: Pictures of Facial Affect^13^, The Yale Face Database^65^, the Karolinska Directed Emotional Faces (KDEF)^15^, the Montreal Set of Facial Displays of Emotion (MSFDE)^66^, the NimStim Set of Facial Expressions^14^, FACES stimulus set^67^, The Warsaw Set of Emotional Facial Expression Pictures (WSEFEP)^68^, The Racially Diverse Affective Expression (RADIATE) stimulus set^69^, The Tsinghua facial expression database (Tsinghua-FED)^70^. Approval for use of these images was obtained from corresponding authors of these databases.

From these databases we selected adult faces to represent the seven basic emotional categories (153 happiness, 148 sadness, 133 anger, 124 surprise, 136 fear, 136 disgust, and 170 neutral). To mirror the WFD, each discrete facial identity was only used once in the ED database. Each database differed in how their images were originally cropped and sized, which meant that some processing was required to ensure all images were consistent with those of the WFD. Where possible, images were resized or cropped to a square shape (400 × 400 pixels). Images with uneven aspect ratios (PoFA, FACES, KDEF, MSFDE, Tsinghua, and WSEFEP) were placed on a uniform grey background (#6e6e6e). Images from The Yale Database were selected for best face visibility (given the set’s emphasis on lighting differences) and faces with sunglasses were included^65^. No ‘calm’ images were selected from the NimStim^14^ Set of Facial Expressions.

#### 10 k US Adult Faces Database

One thousand images were randomly selected from the 10 k US Adult Faces Database^43^ using a random draw without replacement. This database was originally compiled using approximately 25,000 first and last name pairs from a database of names from the United States census, balanced for gender, age and diversity. We first filtered the images for celebrities, then resized the images to 400 pixels in height before placing the images on a square grey canvas.

### Behavioral experiment (remote collection)

#### Participants

In line with previous experiments that have sought to describe visual content in ambient images^38^ we aimed to collect 15–20 ratings per image (*N*_images_ = 3000). We recruited a total of 204 adult, undergraduate students from the University of Queensland to participate in this experiment. Participants completely the experiment remotely and were compensated for their time with a $20 gift voucher. All procedures were approved by The University of Queensland Health and Behavioural Sciences, Low and Negligible Risk Ethics Sub-Committee approved the experimental protocol. Participation was voluntary and anonymous, and every participant provided informed consent.

#### Experimental procedure

The 3000 stimulus faces were randomly assigned to one of 20 subsets of stimuli; each subset was comprised of 150 images (50 WFD faces, 50 ED faces and 50 US faces). We used PsychoPy and Pavlovia to build and host the experimental task; we generated 20 distinct online task links, one for each of the 20 image subsets. Every participant was asked to complete two to three image subsets. We counterbalanced which links were given to a participant, such that every participant completed a unique combination.

For a schematic of the trial structure see Fig. 1C. During the task, participants were presented with an image for 500 ms. This presentation was followed by a screen that asked, “What was the emotion you just saw?”. Underneath the question were nine choices: anger, fear, disgust, sadness, neutral, happiness, surprise, other or unknown. The instructions were to select ‘other’ if they recognized the emotion, but it was not on the list provided, and ‘unknown’ if they did not recognize the emotion. Participants responded in their own time using key presses. Once a selection was made, the next screen asked, “How intense was the emotion?” Intensity ratings were registered by mouse click on a horizontal sliding scale with nine ticks and ‘Not intense at all’ on the far left, and ‘Extremely intense’ on the far right. After this response the participants were asked, “How genuine was the emotion?” Genuineness ratings were registered on a similar sliding scale with ‘Not genuine at all’ on the left, and ‘Extremely genuine’ on the right. After this third response, the trial was complete and there was a 300-ms inter-trial interval before the next trial began. During the inter-trial interval, a central fixation cross was presented. All participants completed seven practice trials at the start of the task before the 150 experimental trials began.

## Data Availability

All materials and data are available via the Open Science Framework https://osf.io/6p4r7/.
